# Cardiac pH-Imaging With Hyperpolarized MRI

**DOI:** 10.3389/fcvm.2020.603674

**Published:** 2020-11-05

**Authors:** Nikolaj Bøgh, Esben Søvsø Szocska Hansen, Christian Østergaard Mariager, Lotte Bonde Bertelsen, Steffen Ringgaard, Christoffer Laustsen

**Affiliations:** The MR Research Center, Department of Clinical Medicine, Aarhus University, Aarhus, Denmark

**Keywords:** magnetic resonance imaging, hyperpolarization, pH, acid-base, heart, myocardium

## Abstract

Regardless of the importance of acid-base disturbances in cardiac disease, there are currently no methods for clinical detection of pH in the heart. Several magnetic resonance imaging techniques hold translational promise and may enable *in-vivo* mapping of pH. We provide a brief overview of these emerging techniques. A particular focus is on the promising advance of magnetic resonance spectroscopy and imaging with hyperpolarized ^13^C-subtrates as biomarkers of myocardial pH. Hyperpolarization allows quantification of key metabolic substrates and their metabolites. Hereby, pH-sensitive reactions can be probed to provide a measure of acid-base alterations. To date, the most used substrates are [1-^13^C]pyruvate and ^13^C-labeled bicarbonate; however, others have been suggested. In cardiovascular medicine, hyperpolarized magnetic resonance spectroscopy has been used to probe acid-base disturbances following pharmacological stress, ischemia and heart failure in animals. In addition to pH-estimation, the technique can quantify fluxes such as the pivotal conversion of pyruvate to lactate via lactate dehydrogenase. This capability, a good safety profile and the fact that the technique is employable in clinical scanners have led to recent translation in early clinical trials. Thus, magnetic resonance spectroscopy and imaging may provide clinical pH-imaging in the near future.

## Introduction

The acid-base balance in the myocardium is firmly regulated within a pH-range of 7.1–7.4, with the cardiomyocytes slightly more acidic (~7.2) than the extracellular space (1). Buffer systems are the first defense against a change in pH. Among these, the histidyl dipeptides seem particularly important (1). Further acid-base regulation is dependent on a range of membrane transporters and intracellular systems. For example, the carbonic anhydrase facilitates intracellular pH-regulation through a variety of mechanisms (1). Acid-base disturbances, both spanning the entire cell or in local microdomains, can cause dysfunction of metabolism, calcium handling and contraction (1). Thereby, improper myocardial pH is associated with inflammation, arrhythmia and ischemia (2–4). While systemic acid-base disturbances are often assessed clinically, no methods exist for pH-imaging of the human myocardium yet, despite its potential to allow localized, spatial assessment of a key component of cardiac disease.

While positron emission tomography (PET) and optical imaging techniques have been used for imaging pH in various organs (5–7), pH-imaging of the heart has mostly been performed with magnetic resonance imaging (MRI). A number of pH-sensitive MRI techniques could translate to clinical use. Most of them utilize pH-dependent properties of the spectral domain of the MR-signal. Some require injection of exogenous agents, while others rely on detection of endogenous molecules. In preclinical cardiovascular work, detection of the chemical shift of inorganic, intracellular phosphorus with ^31^P magnetic resonance spectroscopy has been extensively used for probing intracellular pH in perfused hearts (8). *In-vivo*, the technique has proven to estimate pH correctly in skeletal muscle (9). But *in-vivo* investigations of the heart are limited by spectral contamination from 2,3-diphosphoglycerate in the ventricular blood pool, which has proven difficult to resolve (10, 11). Also using ^31^P spectroscopy, pH can be estimated with the exogenous agent 3-aminopropylphosphonate (3-APP), which displays a chemical shift primarily sensitive to extracellular pH (12). In a similar way, pH can be determined with conventional ^1^H spectroscopy after injection of imidazoles (13) or an oral load of histidine (14). Also using proton MRI, pH can be imaged at relatively high resolution using a variety of gadolinium-based agents with pH-dependent relaxation properties (15–18). More recently, intracellular pH-imaging of the brain has been performed without exogenous agents using chemical exchange saturation transfer techniques (19). However, few of the above techniques have been used in the heart. And none have translated to clinical use in any organ. Many are hindered by toxicity concerns and low sensitivity, insufficient spectral resolution, coarse spatial resolution and field inhomogeneity in clinical scanners. For some of the techniques, the pH-dependent parts of the MR-signal changes with temperature or agent concentration, which confounds the pH-estimate.

The achievable signal from a molecule in MRI is directly related to its prevalence and polarization. Polarization is the overweight of nuclei in the lower-energy spin-state that makes their resonance detectable in the first place. As an example, ^13^C is in principle detectable with MRI, but its low natural abundance and polarization makes it practically futile. Recently, hyperpolarization enabled signal amplification of molecules with MR-sensitive nuclei (20). This allows detection of a wide range of previously undetectable molecules, and has paved the way for new pH-imaging probes. In the following, we review the use of hyperpolarized MRI probes for imaging acid-base disturbances in the heart.

## Overview of Hyperpolarization

**Figure 1:**
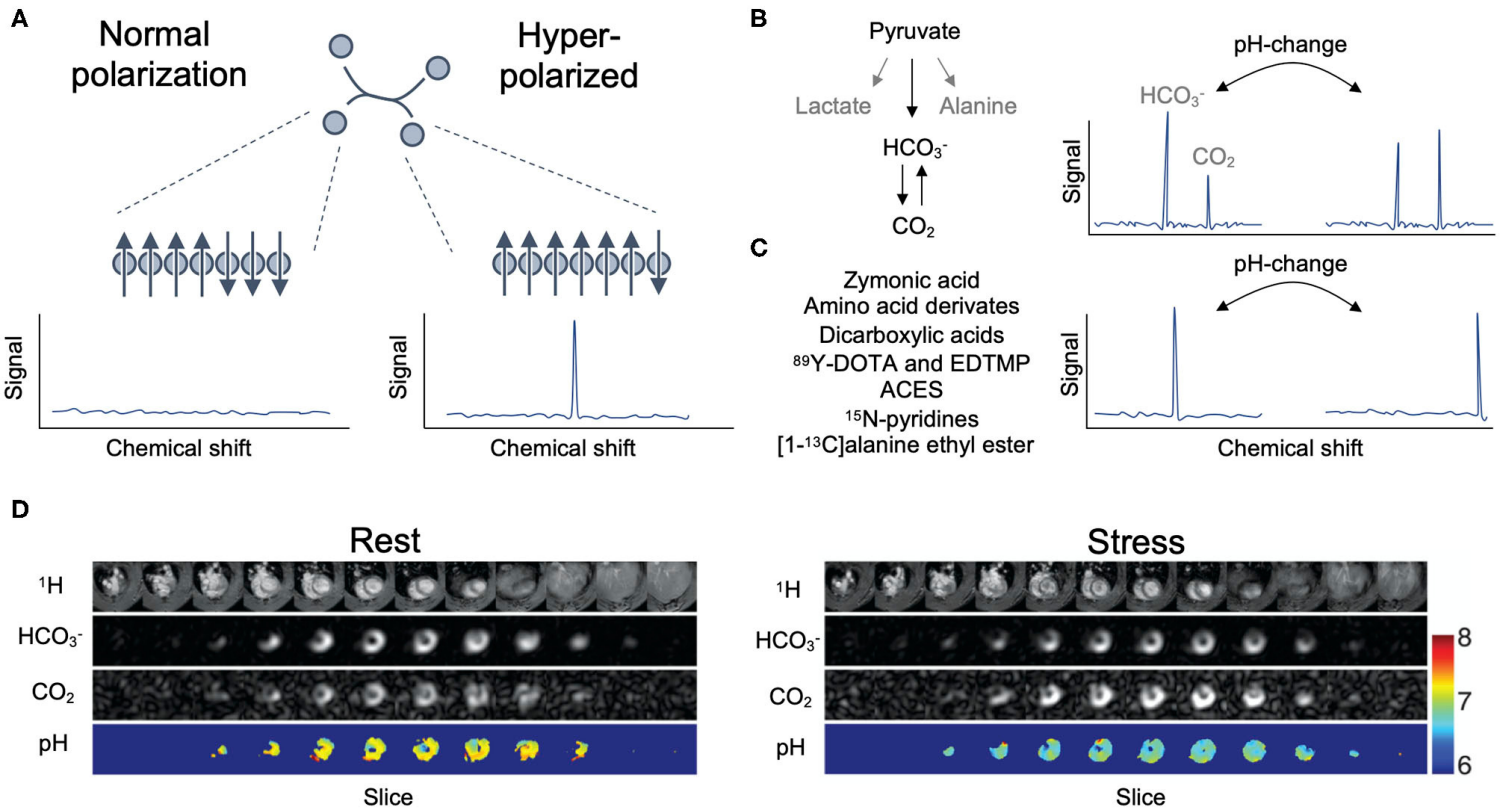
Hyperpolarization enables MR spectroscopy and imaging of otherwise undetectable molecules **(A)**. MR signal origins from abundance and polarization of the nuclei of interest. At low temperatures in a strong magnetic field, polarization can be transferred from electrons to MR sensitive nuclei such as ^13^C by microwave radiation. The polarization of the ^13^C-enriched molecule is increased many-fold over thermal polarization. Thereby, the probe and its metabolites become detectable by spectroscopy after intravenous injection. In hyperpolarized pH-imaging, pH is estimated from the equilibrium between the hyperpolarized acid and its conjugate base. These are either in slow exchange, as is the case with bicarbonate and carbon dioxide after injection of hyperpolarized [1-^13^C]pyruvate or ^13^C-bicarbonate, and pH is estimated by the ratio of their peaks **(B)**. Alternatively, they are in fast exchange, and pH can be estimated from the shift in resonance frequency of a single peak **(C)**. This is the case for hyperpolarized zymonic acid, dicarboxylic acids, amino acid derivates, ^89^Y-marked dodecane tetraacetic acid (DOTA) or ethylenediamine tetramethylenephosphonic acid (EDTMP), *N*-(2-Acetamido)-2-aminoethanesulfonic acid (ACES), ^15^N-pyridine derivatives and [1-^13^C]alanine ethyl ester. Using specialized acquisition strategies, pH can be imaged spatially **(D)**. In this example, the rat heart was imaged *in-vivo* rest and under dobutamine-stress. Hyperpolarized [1-^13^C]pyruvate was used as the probe. This figure is reused and adapted from Lau et al. (21) under an open access Creative Commons CC BY license.

A number of techniques exist for hyperpolarization (20). Dissolution dynamic nuclear polarization is most widely used for pH-imaging *in-vivo* (Figure 1), even though other methods have been employed (22, 23). The technique enables hyperpolarization with commercial equipment (24). The molecule for hyperpolarization is cooled close to ~1 K in a strong magnetic field. Using microwave radiation, the polarization of electrons from a radical is transferred to the target nuclear spin in the molecule. Hereafter, the sample is dissoluted into a solution ready for injection. The technique is most often used for hyperpolarization of ^13^C-enriched molecules, increasing their polarization—and thus signal—way above the thermal equilibrium where conventional MRI is performed. After hyperpolarization, the probe must be injected rapidly due to a swift loss of polarization from relaxation. Then, the hyperpolarized molecule and its metabolites can be detected with spectroscopy or imaging. The most common example is hyperpolarized [1-^13^C]pyruvate, which allows estimation of the glycolytic/oxidative state of an organ through quantification of metabolism of pyruvate to lactate or bicarbonate (25, 26).

## Probing pH With Hyperpolarized MRI

The idea of using hyperpolarized MRI for pH-imaging was coined by Gallagher et al. (27). They imaged a mouse tumor model *in-vivo* with hyperpolarized ^13^C-bicarbonate. Shortly hereafter, Merritt et al. estimated pH from spectroscopy of hyperpolarized [1-^13^C]pyruvate in isolated rat hearts (28). Both utilize a major buffer system of the body by calculating pH from the relative signals from bicarbonate and carbon dioxide. Assuming a pK of ~6.17, the pH can be determined from the Henderson-Hasselbalch relationship:

$$\begin{array}{l} pH=pK_{a}+\log\left(\frac{HCO_{3}^{-}}{CO_{2}}\right) \end{array}$$

This approach assumes high carbonic anhydrase activity for rapid equilibration of the ^13^C-label between bicarbonate and carbon dioxide. With both pyruvate and bicarbonate, estimates of pH are wrong when the carbonic anhydrase is inhibited or absent (27, 29, 30). While myocardial carbonic anhydrase activity is relatively low, Schroeder et al. found that it is sufficient for good pH-estimation in the healthy heart (29). However, mathematical models from the same study suggest that carbonic anhydrase activity may not be sufficient in pathology such as ischemia. Likewise, Merritt et al. found that limited conversion of pyruvate to bicarbonate caused by ischemia hindered estimation of pH (28). Thus, sufficient pyruvate dehydrogenase activity is required when using hyperpolarized [1-^13^C]pyruvate as opposed to ^13^C-bicarbonate. In addition to rapid enzymatic activities and label exchange, the polarization decay of bicarbonate and carbon dioxide are assumed to be similar. They must have comparable spin-lattice relaxation times (T_1_). Merritt et al. measured T_1_ to be 19 s for bicarbonate and 21 s for carbon dioxide at 14.1, while Gallagher et al. found them to be 10.1 and 9.8 s at 9.4 T (27, 28). This small difference between carbon dioxide and bicarbonate can be explained by their rapid interconversion catalyzed by the carbonic anhydrase, and it can be neglected for *in-vivo* measures of pH (27).

Both hyperpolarized ^13^C-bicarbonate and [1-^13^C]pyruvate estimate pH well when carbonic anhydrase activity is sufficient. However, they may estimate the pH in different compartments. It seems that hyperpolarized ^13^C-bicarbonate predominately probes extracellular pH (27). Contrarily, hyperpolarized [1-^13^C]pyruvate pH-estimates are intracellularly weighted (29). This is mainly due to the fact that the pyruvate must first be converted to bicarbonate by the intracellular pyruvate dehydrogenase complex. After conversion, most of the observed signal comes from bicarbonate and carbon dioxide in the cell, as little is transported out for the short duration of the experiment. However, one must be aware that the observed carbon dioxide may partly origin from the extracellular space, as it diffuses freely across the cell membrane. On the contrary to pyruvate, the hyperpolarized ^13^C-bicarbonate is readily available for the high extracellular carbonic anhydrase activity. The injected bicarbonate may not equilibrate fully with the intracellular space during the experiment, further weighting the pH estimate toward the extracellular compartment. However, neither pyruvate or bicarbonate solely estimate one compartment, and their relative weights are not known.

A series of studies have used other hyperpolarized agents than bicarbonate and pyruvate (Table 1). Hyperpolarized [1,5-^13^C_2_]zymonic acid has been shown to have a pH-dependent chemical shift. This allowed Düwel et al. to perform mapping of extracellular pH in rat kidneys and tumors (37). The main advantages include better solubility and longer T_1_ than bicarbonate. Also, the zymonic acid is reportedly non-toxic. Hyperpolarized [1-^13^C]alanine ethyl ester also demonstrates a pH-dependent chemical shift, and no external chemical shift reference is needed (38). It has even better solubility and polarization. As it readily crosses the cell membrane, it allows combined estimation of extra- and intracellular pH. However, the toxicity of alanine ethyl ester remains to be evaluated. Hyperpolarized ^15^N-pyridine derivatives, *N*-(2-Acetamido)-2-aminoethanesulfonic acid (ACES), [2-^13^C,D_10_]diethylmalonic acid, [1-^13^C]serine amide and [1-^13^C]-2,3-diaminopropionic acid have been used for pH-mapping of phantoms through pH-dependent chemical shifts (39–42). In addition to ^13^C and ^15^N, hyperpolarized ^89^Y has been used for pH-imaging. Jindal et al. described the advantageous properties of ^89^Y-marked dodecane tetraacetic acid, while Wang et al. used ^89^Y-marked ethylenediamine tetra methylenephosphonic acid (43, 44). They have a wide chemical shift over physiological pH and relatively long T_1_ values. But, of the molecules described above, only ACES and [1-^13^C]alanine ethyl ester have been evaluated at clinical field strengths, and only hyperpolarized zymonic acid and [1-^13^C]alanine ethyl ester have been applied *in-vivo*.

**Table 1:** Overview of the probes used for pH-imaging with hyperpolarized MRI.

**Probe**	**Nucleus**	**p*K*_**a**_**	**Sensitivity (PPM/pH)**	**Compartment**	**Comment**	**References**
Pyruvate	^13^C	6.17	NA (slow exchange)	Primarily intarcellular	Also allows assessment of metabolism. In clinical translation. Dependent on sufficient PDH and carbonic anhydrase activities.	(21, 28, 29, 31, 32)
Bicarbonate	^13^C	6.17	NA (slow exchange)	Primarily extracellular	Presumably non-toxic after cesium removal. Dependent on sufficient carbonic anhydrase activity.	(27, 30, 33–36)
Zymonic acid	^13^C	6.9	3	Extracellular	Tested *in-vivo* with advantageous MR properties. Reportedly non-toxic.	(37)
Alanine ethyl ester	^13^C	8	~2.2	Both	Good MR properties. Tested *in-vivo*, but toxicity needs to be tested further.	(38)
Pyridine derivatives	^15^N	4.14–7.65	~60	NA	Good MR properties. Not tested *in-vivo*.	(39)
ACES	^13^C	6.58	~4	Extracellular	Good MR properties. Not tested *in-vivo*.	(40)
Amino acid derivates	^13^C	7.35/ 6.95	4.8	NA	Good MR properties. Likely non-toxic, but *in-vivo* testing is warranted.	(41)
Diethylmalonic acid	^13^C	7.39	~2	NA	Long T_1_ and sufficient polarization. Not tested *in-vivo*.	(42)
Dodecane tetraacetic acid	^89^Y	7.64	~3.3	Extracellular	Very long T_1_. Not tested *in-vivo*.	(43)
Ethylenediamine tetramethylenephosphonic acid	^89^Y	6.7	4	Extracellular	Very long T_1_. Not tested *in-vivo*.	(44)
Imidazole	^15^N	6.95	~22.5	NA	Good MR properties. Hyperpolarized with SABRE-SHEATH. Non-hyperpolarized imidazole used *in-vivo* for pH-imaging.	(23)
Encapsulated xenon	^129^Xe	~3	NA	NA	Hyperpolarized gas. Not usable *in-vivo* due to low p*K*_a_	(22)

## Hyperpolarized pH-Imaging of the Heart

Compared to the considerable work performed in cancer, few studies have investigated hyperpolarized MRI for myocardial pH-estimation. Studies of the healthy (21, 45, 46) as well as the diseased (28, 29, 31) heart have been conducted. All except one (46) used hyperpolarized pyruvate. Generally, pH was estimated within the normal range and with good agreement to conventional methods. Due to the relatively low signal-to-noise of the carbon dioxide peak, most studies used spectroscopy of a slice through the ventricles (28, 29, 31, 45). As the heart is much more metabolically active and has larger pyruvate uptake than the surrounding tissue, spectroscopy data of hyperpolarized pyruvate can be assumed to reflect cardiac pH. However, it does not provide information on local acid-base disturbances within the myocardium. In effort to solve this, Lau et al. recently performed pH-imaging (Figure 1D) using spectro-spatial excitation of the bicarbonate and carbon dioxide frequencies (21), thereby paving the way for pH-imaging of the heart.

Only few studies have probed acid-base disturbances in altered physiology or disease. Recently, we confirmed the finding from Lau et al. that hyperpolarized [1-^13^C]pyruvate detects myocardial acidification during increased workload from dobutamine stress (21, 31). In the same paper, we reported that heart failure from right ventricular volume overload decreased myocardial pH *in-vivo*. This is consistent with the involvement of pH-regulating proteins in heart failure (1). Likewise, ischemia-reperfusion injury cause transient myocardial acidosis (1). Accordingly, Schroeder et al. found acidification shortly after ischemia-reperfusion of an isolated rat heart using hyperpolarized pyruvate (29). In a similar model, Merritt et al. were unable to detect changes in pH immediately after ischemia due to low activity of the pyruvate dehydrogenase (28). In both studies, hyperpolarized MRI pH-estimates returned to normal with time. Of interest for cardiac disease, Scholz et al. found that hyperpolarized ^13^C-bicarbonate detected subcutaneous inflammation in a rat model (33). No study has investigated cardiac inflammation, leaving heart failure as the only disease studied *in-vivo*.

## Discussion

Hyperpolarized MRI represents an attractive approach for imaging of acid-base disturbances. Several molecules may be used to probe pH, but as of now, only hyperpolarized pyruvate is approved for use in humans. The ability to image metabolism has driven its lead in the clinical translation. Recently, the first human study of cardiac disease found impaired myocardial energetics with [1-^13^C]pyruvate in diabetic patients (47). Of interest to pH-imaging, they were able to detect carbon dioxide in their spectra. In addition, other hyperpolarized molecules than pyruvate may be approved for human pH-imaging, provided they prove non-toxic. Bicarbonate is promising in this matter, as it is prevalent in the human body. The approach for hyperpolarization originally taken by Gallagher et al. required addition of cesium, which must be removed before injection due to toxicity concerns (27). Later efforts have polarized bicarbonate to similar levels without cesium (34, 46). They hyperpolarized pyruvate or glycerol carbonate, which was then decarboxylated or hydrolyzed to bicarbonate before injection. However, these chemical reactions prolong the critical time from polarization to injection where the hyperpolarization decays. This concern is particularly important for bicarbonate, as it has relatively short T_1_ and low polarization. Added to the required quality control step of clinical trials, the methods of bicarbonate hyperpolarization that require chemical reactions or ion-exchange filtration may extend time-to-injection beyond the acceptable limit. In addition, the relative weights of the extra- and intracellular compartments in pH-estimates with pyruvate and bicarbonate are unknown. This could be investigated with extracellular relaxing agents. Among the other hyperpolarized molecules for pH-imaging, several show attractive properties. Of these, ^89^Y-marked probes are promising due to their long T_1_ (~90 to 120 s) and wide chemical shift albeit relatively low polarization levels (43, 44). Hyperpolarized zymonic acid and [1-^13^C]alanine ethyl ester have been demonstrated *in-vivo* and show better solubility, slightly larger polarization and longer T_1_ than bicarbonate (37, 38). Importantly, these probes do not require detection of a peak with low signal such as carbon dioxide, and they are not flawed in the case of low carbonic anhydrase activity. Those factors limit the use of hyperpolarized pyruvate and bicarbonate to some extent (27, 29, 30). However, most of the alternative probes are not prevalent in the body, and more work is needed to elucidate their *in-vivo* use and toxicity at the relatively high doses needed. As such, hyperpolarized [1-^13^C]pyruvate is the most mature hyperpolarized pH-imaging probe, while the remaining need considerable work before being used in humans.

Even though hyperpolarized [1-^13^C]pyruvate is used in early clinical trials, several challenges remain to be addressed for successful translation. Hyperpolarization with dynamic nuclear polarization requires a polarizer. And specialized equipment must be added to clinical scanners to enable imaging of the relevant nuclei. At the current level of development, the hardware is not sufficiently reliable for routine clinical use. But considerable advances have been made and more are expected (25). In addition to the hardware, pulse sequences could be optimized. Most work in this area has focused on obtaining metabolic information from [1-^13^C]pyruvate, neglecting the carbon dioxide resonance necessary for pH-calculation. As one of the few studies aiming to improve hyperpolarized pH-imaging, Korenchan et al. reported a significant signal-to-noise increase for hyperpolarized ^13^C-bicarbonate using multi flip angle echo-planar imaging (35). In a similar manner, Lau et al. optimized pH-imaging of hyperpolarized [1-^13^C]pyruvate (21). But with their design, no other resonances than bicarbonate and carbon dioxide were detected, and none of the potential metabolic information was obtained. An alternative approach was taken by Drachman et al. (46) who used chemical shift imaging. This allowed detection of all downstream metabolites and thereby both pH-estimation and metabolic assessment. Here, slightly coarser resolution was obtained and the full temporal dynamics could not be captured. Future investigations should determine the optimal trade-offs for sequence design, preferably allowing detection of both acid-base disturbances and metabolic alterations. Alternatively, co-polarization of bicarbonate and pyruvate could provide similar results, even though this is likely to move the weighting of the pH-estimate toward the extracellular pH (45, 48). Both approaches may maximize the clinical potential by adding metabolic information to pH-imaging.

Apart from technical developments, one critical question remains: Where does pH-imaging fit in the clinic, and which patients may benefit from the technique? Only three studies have investigated the technique in disease models (28, 29, 31). More are needed to elucidate its potential across the field of cardiology. Particularly, more *in-vivo* work is warranted. Due to the logistics of MRI in general and hyperpolarized imaging in particular, the technique is most likely to be offered to the patients with chronic illness who are commonly offered cardiac MRI today. Future studies should focus on heart failure, inflammation or coronary artery disease. Even though intra- and extracellular pH are expected to correlate, the different weighting of the probes may be preferential in different diseases. Intracellularly weighted probes are likely to be most valuable in heart failure, as primarily the intracellular pH-changes cause contractile dysfunction and arrhythmia (2, 3). On the contrary, extracellular probes may be more sensitive in the case of ischemia or inflammation, where extracellular acidification seems to be the key player (49, 50). Some diagnostic insight may be gained from hyperpolarized pH-imaging, and even more if it is combined with metabolic assessment using hyperpolarized pyruvate. But the technique may have even more to offer in prognostication, patient selection and early treatment evaluation. For example, from the field of oncology, metabolic assessment with hyperpolarized MRI is reported to correlate with tumor grade (36, 51, 52) and depict the early metabolic response to chemotherapy (32, 53, 54). In cardiology, hyperpolarized MRI could be useful for selecting patients for novel metabolic or pH-altering therapy in heart failure (1), or for detecting the severity, reversibility and localization of ischemia in coronary artery disease. Or it may aid early evaluation of treatment in cardiomyopathies and myocarditis. These exemplify the clear potential of pH and metabolic imaging with hyperpolarized MRI. However, more work in animal models of disease is needed to find the most promising indications and guide design of clinical trials.

In conclusion, MRI with hyperpolarized molecules accurately depicts myocardial acid-base disturbances. While phosphorus MR spectroscopy has long been the technique of choice for measuring pH in perfused hearts, hyperpolarization may expand this capability to clinical MRI. Extracellularly or intracellularly weighted pH-estimates can be obtained with a variety of probes. The most developed probe, [1-^13^C]pyruvate, is under investigation in several clinical trials for its metabolic information, allowing rapid initiation of pH-imaging trials. But, while technical developments are warranted, the most important hurdle is preclinical identification and exploration of the indications where patients may benefit the most.

## Author Contributions

NB wrote the first draft, which was revised by the remaining authors. All authors made a substantial contribution to conceptualization and writing and agree to be accountable for the content of this review.

## Conflict of Interest

The authors declare that the research was conducted in the absence of any commercial or financial relationships that could be construed as a potential conflict of interest.
